# Managing the negative impact of workforce diversity: The important roles of inclusive HRM and employee learning-oriented behaviors

**DOI:** 10.3389/fpsyg.2023.1117690

**Published:** 2023-02-09

**Authors:** Jiakun Liu, Yufeng Zhu, Huatian Wang

**Affiliations:** ^1^Shandong Youth University of Political Science, Jinan, China; ^2^University of International Business and Economics, Beijing, China; ^3^Lingnan University, Hong Kong, Hong Kong SAR, China; ^4^Eindhoven University of Technology, Eindhoven, Netherlands

**Keywords:** workforce diversity, interpersonal conflict, positive and negative affect, inclusive human resources management, employee learning-oriented behaviors

## Abstract

The workforce has become more diverse than it used to be. Although organizations actively capitalize on workforce diversity to enhance team innovation and organizational performance, it is found that workforce diversity also has potential risks, among which interpersonal conflict is the most salient one. However, we still know relatively less about why workforce diversity may link to higher interpersonal conflict and, more importantly, how to mitigate the negative impact of workforce diversity. Based on the workplace diversity theories (e.g., the categorization-elaboration model), this study examined how workforce diversity was positively related to interpersonal conflict through impacting one’s affective states, and to what extent this indirect effect can be weakened by organization-initiated practices (i.e., the inclusive human resources management (HRM) practices) and employee-initiated behaviors (i.e., employee learning-oriented behaviors). Using two-wave surveys from 203 employees from various organizations in China, we confirmed our hypotheses. Our results showed that perceived workforce diversity was positively related to interpersonal conflict through increasing negative affect (after we controlled for the objective diversity level calculated by the Blau index), and this indirect effect was weakened when the levels of inclusive HRM practices and employee learning-oriented behaviors were high. Our study suggests that it is important for organizations to be aware of the detrimental impact of workforce diversity. In addition, it is essential to use both the top-down (e.g., inclusive HRM practices) and bottom-up (e.g., employee learning-oriented behaviors) approaches to managing the challenges presented by diversity so as to unlock more potential of diversity in the workplace.

## Introduction

With the development of globalization and digitalization, contemporary organizations value diverse talents (Chaudhty et al., 2021). They proactively formulate a diverse workforce to boost knowledge integration, product innovation, and organizational overall performance (Roberge and van Dick, 2010; Bell et al., 2011; Díaz-Fernández et al., 2016). Workforce diversity refers to differences based on any characteristics (e.g., age, gender, race, educational and functional backgrounds) on which employees differ or perceive themselves to be different from other co-workers (Guillaume et al., 2017). A European Union survey (2015) shows that 83% of European companies have diverse employees holding different age, gender, and ethnicity, aiming to boost organizational innovation and inclusion. In Asia, A PwC survey (2016) reported that 88% of employers had aligned their diversity and recruitment strategies. Among them, 71% of employers are actively trying to recruit more females, higher than their global counterparts.

Although research has established the positive relationships between diversity and team-level and organizational-level outcomes (e.g., team innovation, team learning, team decision-making, and organizational success; Roberge and van Dick, 2010; Bell et al., 2011; Qian et al., 2013), diversity management is still faced with many challenges. Diversity researchers pointed out that diversity has potential risks. One of the most salient risks is interpersonal conflict (van Knippenberg and Schippers, 2007; Shemla et al., 2016; Wang, 2022). This is because people may make inaccurate judgments about those with different identities and backgrounds, and feel uncertain and distrustful during interpersonal interaction (Van Dick et al., 2008; van Dijk, 2022). As a result, diverse people are more likely to have disagreements and misunderstandings, which finally turn into interpersonal conflicts. Thus, besides pursuing the beneficial outcomes of workforce diversity, a more important question is how to mitigate potential interpersonal conflicts caused by workforce diversity.

While previous diversity management studies uncovered some means of managing diversity in the workplace, such as a supportive team climate (Roberge and van Dick, 2010), team identification (Van Dick et al., 2008), inclusive leadership (Ashikali et al., 2020), and transformational leadership (Kearney and Gebert, 2009), we argue that managing diversity should not just focus on team conditions and leaders’ roles. Other approaches, such as a diversity-focused human resources management (HRM) module and employee-level proactive behaviors, are also important but lack sufficient attention from previous studies (with only a very limited number of studies examining the role of HRM and employee behaviors; see, Hur, 2020; Wang et al., 2022). To fill up this void, this study first empirically examined how workforce diversity was positively linked to interpersonal conflict through impacting one’s affective states (i.e., positive affect and negative affect). Then, based on the diversity theories (e.g., the categorization-elaboration model; van Knippenberg and Schippers, 2007) and the diversity management literature (Shore et al., 2011; Kundu and Mor, 2017; Wang, 2022), we took a top-down as well as a bottom-up perspective to investigate to what extent the impact of workforce diversity on interpersonal conflict through affective states can be alleviated by the inclusive HRM practices and employee self-initiated learning behaviors.

This study aimed to contribute to the diversity literature in several ways. First, by examining the association among workforce diversity, affective states, and interpersonal conflict, we provided empirical evidence on the negative impact of workforce diversity. Thus, we echo the diversity literature (Shemla et al., 2016; Guillaume et al., 2017) by highlighting that diversity is a “double-edged” sword and that the potential risk of workforce diversity should be noticed by organizations and employees. Second, by examining the moderating role of inclusive HRM practices, we provided an effective means to mitigate the negative impact of workforce diversity. We highlight that such a diversity-oriented HRM initiated by organizations is important to unlock the potential of workforce diversity. We contribute to the diversity management literature (Randel et al., 2018; Kundu et al., 2019) by suggesting that inclusive HRM practices including fairness, openness, co-operativeness, support, and empowerment can effectively alleviate the social categorization process in a diverse workplace. Third, by examining the moderating role of employee learning-oriented behaviors, we highlight the important role of employee bottom-up behaviors in managing a diverse work environment. We argue that diversity management is not only a matter of organizations and organizational leaders. Employees themselves can have the autonomy to proactively make use of the benefits of diversity and overcome its detriments (Wang, 2022). Our study suggests that employee learning-oriented behaviors can be such a bottom-up strategy by which employees can facilitate information exchange with different colleagues, increase mutual understanding, and decrease social bias and discrimination. Thus, we add to the diversity management literature (Randel et al., 2018; Kundu et al., 2019; Wang et al., 2022) by underscoring the important role of employee bottom-up behaviors (i.e., learning behaviors) in making better use of workforce diversity. Finally, our study provided empirical evidence on the impact of diversity and the top-down/bottom-up approach to managing diversity in the Asian context, which answered the call from the review study of Chen et al. (2021). We drew more attention to a deeper understanding of the diversity issue in the Asian context, including how and when diversity can function better for Asian organizations and employees.

## Literature review

### Workforce diversity and interpersonal conflict

Workforce diversity refers to differences based on any characteristics (e.g., age, gender, race, educational and functional backgrounds) on which employees differ or perceive themselves to be different from other co-workers (Guillaume et al., 2017). Although people first may relate workforce diversity to racial diversity (i.e., employees who have different nationalities and racial backgrounds) (Singh et al., 2013), diversity researchers have highlighted that diversity has more attributions and actually is very common in the current workplace (Guillaume et al., 2017; Chen et al., 2021; Wang, 2022). For example, nowadays, workplace diversity becomes more age-diverse (e.g., younger generation vs. old generation), gender-diverse (e.g., more females in the C-suite), and functional-diverse (e.g., employees with different functional roles work together for attaining shared goals). To advance the understanding of workforce diversity, researchers attempted to categorize diversity into surface-level diversity and deep-level diversity (Harrison et al., 2002; Hecht and Allen, 2009). Surface-level diversity refers to observed characteristic differences, such as age, gender, and race; while deep-level diversity refers to differences in terms of one’s personal values, beliefs, and cognitions (Hecht and Allen, 2009). As a result, even if the surface-level diversity is low (e.g., people are in a similar age range), deep-level diversity can be high (e.g., people are different in their personal values).

The diversity literature uncovered some beneficial effects of workforce diversity on individuals (e.g., creativity and inspiration) (Kim et al., 2020), groups (e.g., group performance and innovation) (Van Knippenberg et al., 2004; Guo et al., 2021; Hou et al., 2021), and organizations (e.g., organizational performance and reputation) (Dwyer et al., 2003). This is because, based on an information processing perspective (Dahlin et al., 2005), people with different backgrounds, demographics, and cognitions can provide a larger resource pool in the workplace. These resources may include different knowledge, skills, and perspectives that can be used to facilitate work processes (Porter and Woo, 2015). Research also suggests that workforce diversity can help to increase organizational competitive advantages, retain diverse talents, and create a more diverse and inclusive work environment (Cox and Blake, 2013; Wang, 2022).

However, diversity researchers also found that workforce diversity may not always be beneficial. It can also lead to detrimental consequences. One of the most salient risks is a higher likelihood of *interpersonal conflict*. Interpersonal conflict refers to interpersonal clashes unrelated to task issues (Jehn and Bendersky, 2003). Research shows that interpersonal conflict contains three important elements, that is, some form of interaction between individuals, interdependence among individuals, and an incompatibility based on perception or values (Zhang and Huo, 2015). When experiencing conflicts wither others, people tend to show mistreatment behaviors, distrust, and work stress (Bruk-lee and Spector, 2006). It is more like to occur when individuals have diverse believes and values (Barki and Hartwick, 2004). The social identity (Roberge and van Dick, 2010) and social categorization (Van Knippenberg et al., 2004) theories state that individuals like to categorize themselves and others in various social groups. People classify those who have similar values, habits, and cognitions as “in-group,” while those who have different values and interests as “out-group” (Van Knippenberg et al., 2004). As a result, individuals may generate bias for “out-group” members, distrust them, and feel uncertain and threatened, which will, in turn, induce interpersonal conflict. Following this reasoning, we can infer that workforce diversity may include employees with different interests, values, and preferences. When working in such a work environment, employees may have a higher chance to have conflicts and disagreements. Below, we take an affective state perspective to understand the intermediate process by which workforce diversity may stimulate interpersonal conflict.

### Positive affect and negative affect

Affective states such as moods or emotions are important in the workplace (Hentschel et al., 2013). Negative affect (NA) reflects emotions subsuming a variety of aversive mood states, including anger, contempt, disgust, guilt, fear, and nervousness, while enthusiasm, inspiration, and appreciation are some examples of positive affect (PA) (Ghasemy et al., 2022). The affective events theory (Weiss and Cropanzano, 1996) suggests that work events, either related to people or situations, can influence emotions and moods which subsequently result in affect-driven behaviors and attitudes. The cognitively-oriented research on emotion indicates that one’s affective reactions are at least in part the outcome of the cognitive appraisal of a situation (Weiss and Cropanzano, 1996; Mignonac and Herrbach, 2004). Every person-environment interaction has a potential emotional significance. Positive emotions arise from interactions that favor the individuals’ values and interests, while negative emotions arise when an interaction is an obstacle to the individual’s goals, needs or values (Mignonac and Herrbach, 2004). Prior review studies on affectivity-performance relationship have confirmed that PA is positively related to job performance, while NA is negatively related to job performance (Kaplan et al., 2009). This is because experiences of positive emotionality can foster the vigor, energy, and excitement that accompany reward-seeking behavior (Watson et al., 1999). Conversely, NA may induce avoidance-type behaviors when individuals encounters potentially threatening or aversive conditions (Watson et al., 1999). Besides, studies found that affective states can influence other job outcomes, such as job satisfaction (Judge and Larsen, 2001), effective leadership (Cropanzano et al., 2003), sales effectiveness (Tsai and Huang, 2002), and creative problem solving (Cropanzano et al., 2003). Finally, research shows that different organizational and personal characteristics can the antecedents of positive and negative affects at work (Kiefer, 2005; Shirom, 2007). For example, empirical evidence showed that organizational change (e.g., working conditions, organizational treatment) can influence one’s affective states, which can further influence withdrawal behaviors and trust (Kiefer, 2005). Shirom (2007) took a resource perspective and pointed out that organizational-level resources (e.g., participation in decision-making; rewords and practices), group-level resources (e.g., supervisor support, colleague support, and cohesiveness), and individual-level resources (e.g., expertise, power, and autonomy) are all important antecedents of one’s affective states.

### Inclusive human resource management practices

The inclusive human resource management (HRM) practice refers to a series of human resource management activities to respect and manage employee differences, enhance fairness and justice, and offer necessary support and empowerment to employees (Lu, 2018; Hur, 2020). The inclusive HRM practice was based on the inclusion theory (Shore et al., 2011; Jansen et al., 2014). The theory states that organizations that value employees’ belonging and uniqueness (i.e., two dimensions of workplace inclusion) can obtain more positive employee outcomes in a diverse workplace (Shore et al., 2011). This is because employees can feel respected for their identities and be treated equally when organizations foster inclusion. As a result, they will have a higher level of organizational commitment and work engagement (Hur, 2020; Chaudhty et al., 2021). Thus, based on the inclusion literature, inclusive HRM practices include many inclusion-based components: respecting and managing employee differences, enhancing fairness and justice, and offering necessary support and empowerment to employees (Lu, 2018; Hur, 2020). Hur (2020) described that creating an inclusive work environment included five basic practices: fairness, openness, co-operativeness, support, and empowerment. In line with the western inclusive HRM practices (e.g., age-diverse inclusive HRM and inclusive leadership; Ashikali et al., 2020; Oliveira, 2021), recently, in the Asian context, Tang et al. (2015) introduced seven factors of inclusive HRM practices for Chinese organizations including inclusive teamwork (e.g., team-building, information sharing, and divergent thinking), democratic communication, inclusive decision-making (e.g., participation in decision-making, group discussion, and delegation), fairness treatment, inclusive leadership, tolerance (e.g., tolerance mistakes), and inclusive adaptation (e.g., adapting to the organization). These components can help to facilitate employees’ belonging but their uniqueness as well. The workplace inclusion studies found that workplace inclusion can help to reduce uncertainty and insecurity, increase mutual understanding and trust, and enhance work motivation and psychological safety (c.f. Mor Barak et al., 2016; Jaiswal and Dyaram, 2020; van Dijk, 2022). A review study by Mor Barak et al. (2016) indicated that inclusion can buffer the negative impact of workplace diversity (e.g., discrimination, conflicts, and bias). The HRM literature shows that inclusive HRM practices were positively related to inclusive disability organizational climate (Rizky, 2021), employee job satisfaction and affective commitment (Hur, 2020), the thriving of older workers (Oliveira, 2021), and organizational innovation (Chaudhty et al., 2021). Thus, when organizations implement more inclusion-based HRM practices, employees will be able to experience more positive emotions, fewer negative emotions, and fewer conflicts at work or during social interactions.

Notably, inclusion-based HRM practices should also include diversity training, so that employees can gain the actual learning outcomes of diversity mindset and diversity-related behaviors (Smith et al., 2012). While inclusive HRM practices focus more on distal outcomes such as affirmative action plans, diversity-tolerant cultures, diversity management executives (Kalinoski et al., 2013), diversity training is likely to enhance more proximal outcomes (Kulik and Roberson, 2008). Studies showed that diversity training can increase team creativity (Homan et al., 2015), reduce prejudice among students (Rudman et al., 2001), enhance the multicultural skills of nurses and medical students (Smith et al., 2006), improve productivity and engagement of diverse employees, and help retention of women and people of color in the workplace (Anand and Winters, 2008).

### Employee learning-oriented behaviors

Employee learning-oriented behavior refers to discretionary actions in ongoing learning activities to master new knowledge, skills, and abilities (Bezuijen et al., 2009). The goal orientation literature (Heimbeck et al., 2003; Matsuo, 2019) indicates that a learning orientation can facilitate mastery of challenging tasks and deeper engagement with the task (Hirst et al., 2009). When obstacles are encountered, learning-oriented people tend to deal with these challenges by investing additional effort to resolve the problem (Hirst et al., 2009) and are likely to cope effectively with both negative and positive feedback by identifying and applying more creative problem-solving activities (Dweck, 2013). Thus, learning-oriented people may feel more confident to respond to difficulties and challenges, as well as feel optimistic about their current role or the future (Kim et al., 2015). Maurer (2002) introduced an employee learning orientation model indicating that employee learning may include cognitive constructs (e.g., self-discrepancy), affective constructs (e.g., positive attitudes and affect), and behavioral constructs (e.g., participation and persistence). Research has shown that employee learning-oriented behaviors are positively related to employability (Kim et al., 2015), creativity (Hirst et al., 2009), and job performance (Walumbwa et al., 2009). Employee learning orientation behaviors also delineate a proactive behavioral process of oneself. That is, employees engage in multiple learning activities in a more proactive way that reflects upon their personal needs and goals. The proactivity literature (Crant, 2000) indicates that when one is proactive in their work process, they can gain a higher level of work engagement and are more motivated to seek resources from others (Matsuo, 2019). Learning-oriented behaviors strive to increase their level of competence (Heimbeck et al., 2003). Learning goal orientation was associated with high persistence in the face of failure, which led to higher skill development (Heimbeck et al., 2003).

## Hypothesis development

### The mediating role of positive and negative affects

Based on the positive and negative affectivity literature (Cropanzano et al., 2003) as well as the workforce diversity literature (Van Knippenberg et al., 2004), we argue that workforce diversity may increase the likelihood of interpersonal conflict through impacting one’s affective states (i.e., increasing negative affect and decreasing positive affect). When employees perceive their colleagues to be different from their own goals and interests, they tend to feel more defensive, nervous, and uncertain, as well as less appreciated, assertive, and enthusiastic (i.e., negative affect may arise and positive affect may decline), which in turn, may induce some aggressive behaviors, such as interpersonal conflict. For example, when workgroup members have diverse educational and functional backgrounds, they will be more likely to have different opinions and perspectives, which will take more time to understand and process. This will make people feel exhausted, uncertain, and stressed (Wang, 2022). With such affective states, people may be more inclined to have disagreements, devaluate others’ functional roles, and finally result in interpersonal conflict. Similarly, if employees come from different ethnic and racial backgrounds, they are more likely to have biases and make inaccurate judgements to each other (van Dijk, 2022). This will increase negative affects, which may lead to discrimination and interpersonal conflict. Taking together, we formulate our first hypothesis:

*H1*: Perceived workforce diversity is positively linked to interpersonal conflicts via (a) increasing negative affect and (b) decreasing positive affect.

### The moderating role of inclusive HRM practices

While knowing the mediating role of affective states on the relationship between workforce and interpersonal conflicts, a more meaningful question is under what conditions organizations and employees may alleviate the negative impact of workforce diversity. From a top-down perspective, we propose that an inclusive HRM practice can be an important circumstance that weakens the negative impact of workforce diversity. Based on the diversity theories (e.g., social identity and social categorization theories; Van Knippenberg et al., 2004; Van Dick et al., 2008) and the inclusive HRM literature (Tang et al., 2015; Hur, 2020), we argue that the inclusive HMR practices can create a more inclusive work environment under which people with different backgrounds and identities can fully exchange their ideas and voice their different opinions. For example, people with different educational and functional backgrounds will feel psychologically safe to voice in teams under an inclusive work environment. People of different age, gender, or race may increase mutual understanding and decrease biases under an inclusive work environment. As a result, the impact of workforce diversity on interpersonal conflicts through affective states may be buffered. Research shows that inclusion practices can increase procedural justice, which can strengthen fairness among employees irrespective of their age, gender, race, and social background (Kundu et al., 2019). Shore et al. (2011) highlighted that inclusion is an important factor in managing diversity. Inclusion can make employees perceive more belongingness but also uniqueness (i.e., maintaining a distinctive and differentiated sense of self). Similarly, van Dijk (2022) indicated that without inclusion, people may feel more uncertain, make inaccurate attributions of expertise, and exacerbate the negative consequences of diversity. To conclude, we argue that inclusive HRM practices may effectively alleviate the negative impact of workforce diversity. We hypothesize:

*H2*: The effect of perceived workforce diversity on interpersonal conflicts via (a) increasing negative affect and (b) decreasing positive affect is weakened if organizations implement more inclusive HRM practices.

### The moderating role of employee learning-oriented behaviors

**Figure 1:**
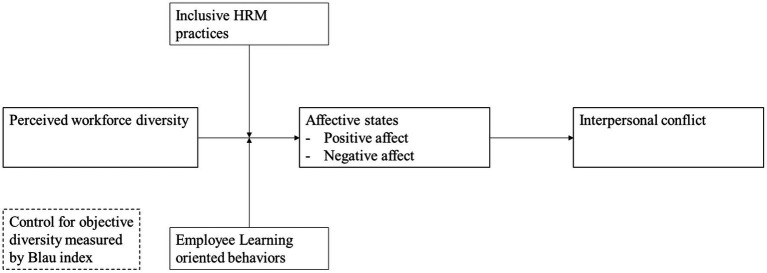
Conceptual model.

In addition to the top-down approach (e.g., inclusive HRM practices) to managing workforce diversity, it is equally important to consider the bottom-up approach. That is, how employees can engage in certain strategies to alleviate the negative impact of workforce diversity on themselves. We propose that employee learning-oriented behavior can be one of the effective bottom-up approaches. Based on the diversity theories (Van Knippenberg et al., 2004; Van Dick et al., 2008) and employee learning literature (Maurer, 2002; Bezuijen et al., 2009), we argue that employee learning-oriented behaviors may help to alleviate the negative impact of workforce diversity. That is, those who are more learning-oriented may perceive diversity as a learning opportunity and try to make use of such differences (e.g., knowledge, skills, and experience differences) among colleagues to facilitate their own tasks. Thus, when employees are learning-oriented, they may frame obstacles presented by diversity as a challenging task and invest additional effort to resolve them (Hirst et al., 2009). They are more likely to mobilize their positive affect and psychological resources to deal with difficulties that may emerge during a learning process. For example, when colleagues have different educational and/or functional backgrounds, those who are learning-oriented are more likely to proactively seek feedback and use others’ expertise to optimize their own work process (Wang et al., 2022). Learning-oriented employees may be more motivated to learn and understand differences among colleagues, and create meaningful conversations with one another, which can effectively reduce bias and misunderstanding. Thus, we argue that employee learning-oriented behaviors can be one of the effective bottom-up approaches to minimize the negative impact and maximize the positive impact of workforce diversity (Figure 1). We hypothesize:

*H3*: The effect of perceived workforce diversity on interpersonal conflicts via (a) increasing negative affect and (b) decreasing positive affect is weakened when employees engage in more learning-oriented behaviors.

## Methods

### Sample and participants

We collected data from three medium-sized service organizations including one education institute, one estate company, and one property management company in Shandong, China. The first and second authors engaged with the managers of these organizations to seek for cooperation. We used the online survey platform Wenjuanxing to send the questionnaires. The consent form needs to be completed before filling in the questionnaires. Participants were assured that their answers would be treated confidentially, and their responses were anonymous. The questionnaires were sent in two times separately. The first questionnaire measures participants’ demographic information (e.g., age, gender, tenure, educational background, and occupation), predictor (i.e., perceived workforce diversity) and moderator (i.e., inclusive HRM practices and employee learning-oriented behaviors). At the end of the first questionnaire, participants were asked to create a unique identification code, so that we can match them with their second questionnaire. Besides, we also asked participants to fill in their leaders’ email addresses. We then sent another survey to their leader to evaluate the diversity level (e.g., age, gender, and educational degree diversity) in their work group as we think the leader-rating is more objective. We considered these objective diversity measures as control variables. Two weeks later, we sent the second questionnaire. The second questionnaire measures the mediator (i.e., positive and negative affect) and the outcome (i.e., interpersonal conflicts).

322 participants completed the first questionnaires, among which 222 leaders responded for the objective diversity measures. 203 participants completed the second questionnaire. After matching the two questionnaires using the unique identification code, we finally yield 182 data points. The response rate was 56.52%. Among them, 39.2% were male and 60.8% were female. The average age was 28.71 (SD = 7.23). The average working years were 4.17 (SD = 5.39). 84.23% of them obtain the bachelor’s degree and above.

### Measures

We administered the questionnaires in Chinese and followed the back-translation procedure to ensure consistency (Brislin, 1976). That is, we first translated the items into Chinese. Then we asked another professional linguist (an English teacher) to perform a literal translation of the items back to English. Finally, we compared the back translation to the original text to make sure the back translation is accurate and complete.

*Workforce diversity* was measured using the scales developed by Harrison et al. (2002) and Harrison and Klein (2007). To avoid confusing participants, we consider workforce diversity as perceived diversity in their current work group/department as the current work group/department is the place participants spend most time on. Eight items were included to measure different aspects of perceived diversity (e.g., age, marital status, educational background, personalities, personal values, priorities to do things, expertise, and socially valued assets). An example item was “to what extent do you think the members of your work group/department were similar in terms of age.” The scale ranged from 1 (very similar) to 5 (very different). The Cronbach’s Alpha was 0.892.

*Positive and negative affect* was measured using the scale developed by Liu et al. (2020). Six items measured negative affect (e.g., I feel upset, Cronbach’s Alpha = 0.936), while four items measured positive affect (e.g., I feel inspired, Cronbach’s Alpha = 0.905). The scale ranged from 1 (never) to 5 (always).

*Interpersonal conflict* was measured using the scale developed by Zhang and Huo (2015). Four items were included. An example item was “How much friction is present in your work unit.” The scale ranged from 1 (none) to 5 (a lot). Cronbach’s Alpha = 0.963.

*Inclusive HRM practice* was measured using the four-item scale developed by Chaudhty et al. (2021). In addition, we selected another seven items from the study of Tang et al. (2015) to enrich the content of inclusive HRM practice as these newly added items fit the Chinese context. The scale ranged from 1 (very disagree) to 5 (very agree). Cronbach’s Alpha = 0.952. The full items of inclusive HRM practice were displayed in the Appendix.

*Employee learning-oriented behavior* was measured using the scale developed by Bezuijen et al. (2010). Eight items were included. An example item was “Within my job, I look for activities from which I can learn.” The scale ranged from 1 (never) to 5 (very often). Cronbach’s Alpha = 0.916.

*Control variables.* Besides controlling for participants’ age and gender, we also calculated the objective diversity (leader-rating) and add it as a control variable. As mentioned earlier, we asked the leader to evaluate the level of diversity in their work unit. Specifically, the leader needs to indicate: (1) how many people in their work unit; (2) among them, how many males and how many females; (3) among them, how many people work less than 5 years, how many people work between 5 and 10 years, and how many people work more than 10 years; (3) among them, how many people have a vocational degree, how many people have a bachelor degree, and how many people have a master degree (and above). By asking leaders to indicate these questions, we used the Blau index ^1^(see, Harrison and Klein, 2007) to calculate work unit gender diversity, tenure diversity, and educational diversity, respectively.

### Analytical approach

We performed structural equation modelling using Mplus to test our hypotheses. We first drew the best-fitting measurement model and then the structural model. To calculate the conditional indirect effect, we used the PROCESS developed by Hayes (2015).

## Results

### Preliminary results

The means, S.D., and correlations among studied variables were shown in Table 1. Regarding the measurement model, we tested several models and selected the best fitting one. See Table 2. We compared different measurement model. The results indicated that model 1 was the best fitting one. The results showed that model 1: the six-factor model [*χ*^2^(786) = 1373.021; CFI = 0.915; SRMR = 0.067; RMSEA = 0.058] was significantly better than five-factor model [*χ*^2^(791) = 1692.277; CFI = 0.869; SRMR = 0.106; RMSEA = 0.072; Δ*χ*^2^ (5) = 319.26, *p* < 0.001], and that it’s even significantly better than four-factor model [*χ*^2^(795) = 1906.17; CFI = 0.839; SRMR = 0.108; RMSEA = 0.079; Δ*χ*^2^ (9) = 533.15, *p* < 0.001]. Therefore, we conclude that our focal variables are differentiated from each other, and that the discriminant validity can be confirmed.

**Table 1:** Means, SDs, and correlations among studied variables (*N* = 222).

	Mean	S.D.	Workforce diversity_T1	Inclusive HRM practices_T1	Employee learning behaviors_T1	Positive affect_T2	Negative affect_T2	Interpersonal conflicts_T2	Gender diversity_T1	Tenure diversity_T1	Education degree diversity_T1	Age_T1	Gender_T1
Workforce diversity_T1	2.626	0.881											
Inclusive HRM practices_T1	3.842	0.698	−0.083										
Employee learning behaviors_T1	3.785	0.648	0.013	0.672**									
Positive affect_T2	3.757	0.743	−0.028	0.372**	0.436**								
Negative affect_T2	2.401	0.956	0.214**	−0.243**	−0.082	0.046							
Interpersonal conflicts_T2	2.327	0.986	0.183*	−0.188*	−0.089	0.100	0.759**						
Gender diversity_T1	0.391	0.147	0.111	0.006	0.031	−0.013	0.108	−0.018					
Tenure diversity_T1	0.493	0.235	0.215**	−0.180**	−0.130	−0.092	0.118	0.085	0.214**				
Education degree diversity_T1	0.387	0.222	0.153*	−0.115	−0.116	−0.027	0.169*	0.151*	0.229**	0.229**			
Age_T1	28.71	7.233	−0.032	−0.014	−0.006	−0.050	−0.096	0.006	0.012	−0.018	0.007		
Gender_T1	1.61	0.489	0.036	−0.065	−0.124	−0.126	−0.183*	−0.238**	0.026	0.120	0.005	−0.141*	

**p < 0.01; *p < 0.05 level (2-tailed). T1 refers to variables measured at time 1; T2 refers to variables measured at time 2.

**Table 2:** Results of confirmatory factor analyses (measurement model).

Models	*χ*2	df	RMSEA	CFI	SRMR	Compare mode differences
1. Six-factor model	1373.021	786	0.058	0.915	0.067	
2. Five-factor model	1692.277	791	0.072	0.869	0.106	Model 1 vs. Model 2: Δ*χ*^2^ (5) = 319.26, *p* < 0.001
3. Four-factor model	1906.170	795	0.079	0.839	0.108	Model 1 vs. Model 3: Δ*χ*^2^ (9) = 533.15, *p* < 0.001
4. Three-factor model	2252.675	798	0.091	0.789	0.117	Model 1 vs. Model 4: Δ*χ*^2^ (12) = 879.65, *p* < 0.001

Six-factor model includes: perceived workforce diversity, positive affect, negative affect, interpersonal conflict, inclusive HRM practice, employee learning behavior; five-factor model includes: collapsing positive affect and negative affect into one factor; four-factor model includes: collapsing positive affect, negative affect, and interpersonal conflict into one factor; three-factor model: collapsing positive affect, negative affect, and interpersonal conflict into one factor, as well as collapsing inclusive HRM practice and employee learning behavior into one factor.

**Figure 2:**
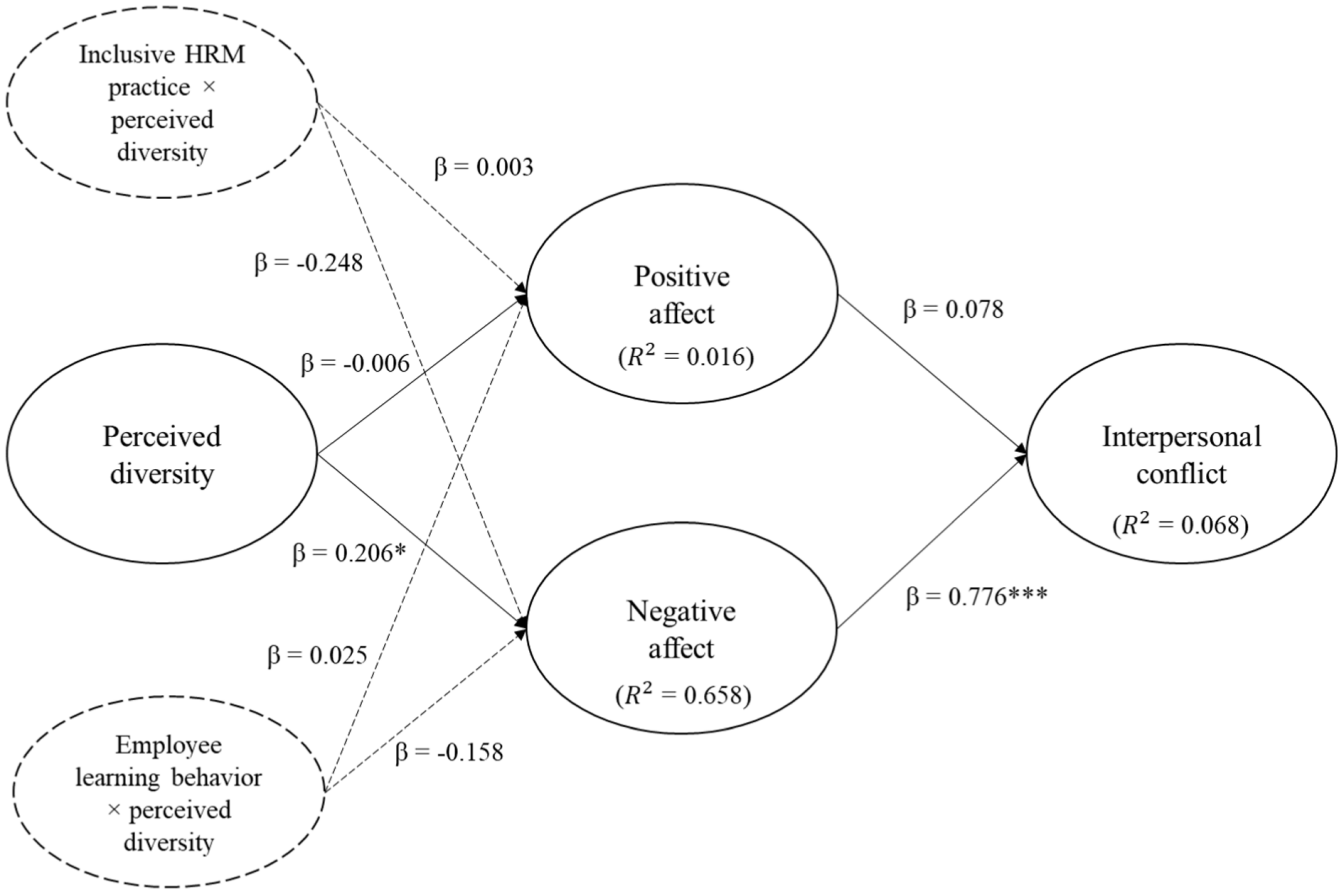
Path regression results using the SEM approach. Standardized coefficients (*β*) are reported; *N* = 222; ***p < 0.001, **p < 0.01, *p < 0.05. Model fit: Chi-Square = 1387.307; df = 791; RMSEA = 0.058; CFI = 0.913; TLI = 0.906; SRMR = 0.07.

To assess the structural model, we found that the *R*^2^ for positive affect, negative affect, interpersonal conflict, inclusive HRM practice, and employee learning behavior was 0.016, 0.658, 0.068, 0.25, and 0.263, respectively, (See Figure 2). We also calculated the predictive relevance (*Q*^2^ = 1 – SSE/SSO). The results showed that the predictive relevance (*Q*^2^) was 0.118, 0.05, and 0.519 for positive affect, negative affect, and interpersonal conflict, respectively. To conclude, the structural model was also acceptable in this study.

### Common methods bias (CMB)

The Harman’s one-factor results showed that there was 27.2% of variance extracted, which was less than the threshold of 50%. However, since this technique got some criticism for its insufficient sensitivity to detect moderate or small levels of CMV effects (Malhotra et al., 2006), we did another test – unmeasured latent method factor analysis (Podsakoff et al., 2011) using Mplus (also see, Wang et al., 2022) – to replicate Harman’s one-factor test results. We found that the unconstrained model fit was *χ*^2^ = 1396.762, df = 762; while the constrained model fit was *χ*^2^ = 1670.23, df = 795. The model comparison was Δ*χ*^2^ (33) = 273.47, *p* < 0.001. This implies that there exists potential common method bias. We further examined how much variance was shared among all of the items to see if the common method bias is a serious issue. We conducted the equal constrained model. The results showed that the potential “method” factor shared the variance of 9.49%. Thus, we concluded that CMB might not be a serious issue in this study.

### Hypotheses testing

We first examined the indirect effect of diversity on conflict *via* positive and negative effects. The results (in Table 3) showed that the indirect effect of diversity on conflict *via* negative affect was significant (*b* = 0.16, [0.005, 0.311]), while the indirect effect *via* positive affect was nonsignificant (*b* = −0.007, [−0.017, 0.013]). Thus, hypothesis 1a was supported but 1b was not supported.

**Table 3:** Process results of indirect effects and conditional indirect effects.

Control for gender diversity, tenure diversity, and educational degree diversity			*b*	se	LLCI	ULCI
Indirect effects						
Perceived workforce diversity → negative affect → interpersonal conflicts			0.16	0.08	**0.005**	**0.311**
Perceived workforce diversity → positive affect → interpersonal conflicts			−0.007	0.007	−0.017	0.013
*Conditional indirect effects*						
Inclusive HRM practices	−1SD	Perceived workforce diversity → negative affect → interpersonal conflicts	0.301	0.109	**0.088**	**0.523**
		Perceived workforce diversity → positive affect → interpersonal conflicts	0.002	0.012	−0.024	0.026
	+1SD	Perceived workforce diversity → negative affect → interpersonal conflicts	0.077	0.102	−0.129	0.276
		Perceived workforce diversity → positive affect → interpersonal conflicts	0.002	0.008	−0.019	0.017
Employee learning behaviors	−1SD	Perceived workforce diversity → negative affect → interpersonal conflicts	0.265	0.112	**0.055**	**0.497**
		Perceived workforce diversity → positive affect → interpersonal conflicts	−0.005	0.013	−0.033	0.022
	+1SD	Perceived workforce diversity → negative affect → interpersonal conflicts	0.126	0.098	−0.069	0.315
		Perceived workforce diversity → positive affect → interpersonal conflicts	−0.003	0.008	−0.021	0.009

*N* = 222 data points, and *N* = 182 matched data points. Bootstrapping iterations are 5,000. LLCI, lower level confidence intervals; ULCI, upper level confidence intervals. The bold values represent the significant confidence interval.

To examine the conditional indirect effects of inclusive HRM practice and employ learning behavior, we used PROCESS analyses. The results (Table 3) showed that the negative indirect effect of diversity on conflict *via* negative affect became weaker when organizations engaged in more inclusive HRM practice (*b* = 0.301, [0.088, 0.523]). But the conditional indirect effect of inclusive HRM practice was nonsignificant for the positive affect. Also, we confirmed that the negative indirect effect of diversity on conflict *via* negative affect became weaker when employees engaged in more learning-oriented behaviors (*b* = 0.265, [0.055, 0.497]). But the conditional indirect effect of employee learning behaviors was nonsignificant for the positive affect. Thus, hypothesis 2a and 3a were supported, while 2b and 3b were not supported.

**Figure 3:**
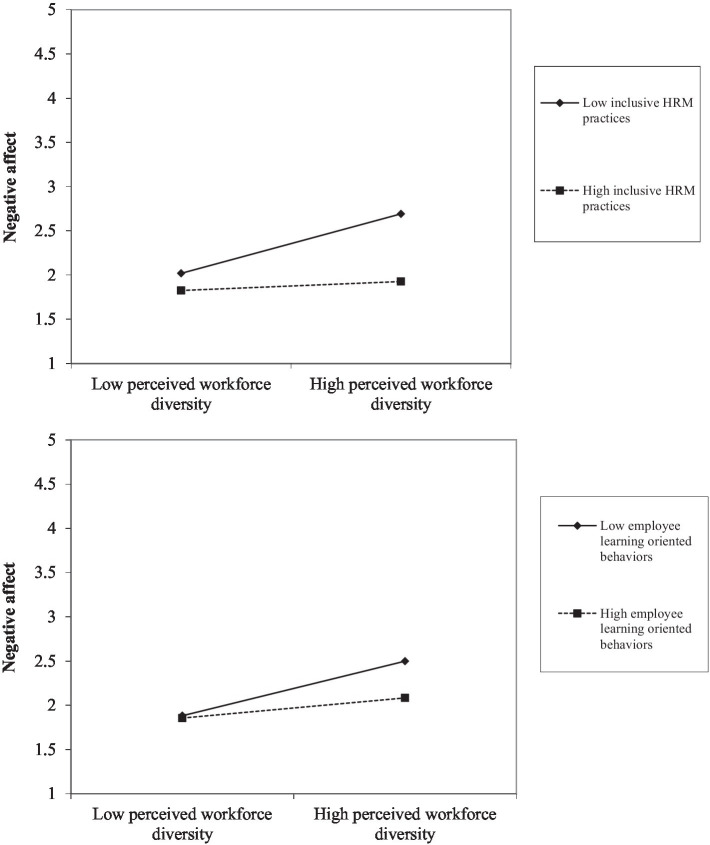
**(A)** The two-way interaction between workforce diversity and inclusive HRM practices on employee negative affect. **(B)** The two-way interaction between workforce diversity and employee learning behaviors on employee negative affect.

Finally, we plotted the two-way interaction figures to visualize the moderating role of inclusive HRM practice and employee learning behavior, as shown in Figures 3A,B. The simple slope test showed that perceived workforce diversity was only negatively related to negative affect when the level of inclusive HRM practice was low (*b* = 0.382, *p* = 0.003), while this detrimental impact was nonsignificant if the level of inclusive HRM practice was high (*b* = 0.058, *p* = 0.59). Similarly, perceived workforce diversity was only negatively related to negative affect when the level of employee learning behavior was low (*b* = 0.35, *p* = 0.014), while this detrimental impact was nonsignificant if the level of employee learning behavior was high (*b* = 0.13, *p* = 0.23). Please check Factor loadings, CR, and AVE for the studied variables below (Table 4).

**Table 4:** Factor loadings, CR, and AVE for studied variables.

Constructs	Factor loading	CR	AVE	Cronbach’s *α*
Workforce diversity		0.928	0.587	0.892
DIV1	0.669			
DIV2	0.731			
DIV3	0.805			
DIV4	0.745			
DIV5	0.722			
DIV6	0.737			
DIV7	0.839			
DIV8	0.861			
Interpersonal conflict		0.963	0.901	0.963
IC1	0.952			
IC2	0.947			
IC3	0.954			
IC4	0.944			
Positive affect		0.978	0.772	0.905
PA1	0.848			
PA2	0.898			
PA3	0.843			
PA4	0.923			
Negative affect		0.942	0.763	0.936
NA1	0.847			
NA2	0.886			
NA3	0.905			
NA4	0.908			
NA5	0.901			
NA6	0.787			
Inclusive HRM practices		0.962	0.677	0.952
HRM1	0.811			
HRM2	0.783			
HRM3	0.755			
HRM4	0.811			
HRM5	0.828			
HRM6	0.878			
HRM7	0.840			
HRM8	0.868			
HRM9	0.864			
HRM10	0.778			
HRM11	0.826			
Employee learning behaviors		0.789	0.549	0.916
LEARN1	0.511			
LEARN2	0.717			
LEARN3	0.683			
LEARN4	0.733			
LEARN5	0.523			
LEARN6	0.886			
LEARN7	0.886			
LEARN8	0.878			

CR refers to composite reliability; AVE refers to average variance extracted; Cronbach’s *α* refers to items internal consistency.

## Discussion

Workforce diversity is a “double-edged sword” for organizations and employees (Wang, 2022). Based on diversity theories (e.g., social identity and social categorization perspectives; Van Knippenberg et al., 2004; Van Dick et al., 2008), we uncovered how workforce diversity was positively linked to interpersonal conflict through impacting affective states. Our results showed that workforce diversity was positively related to negative affect, which in turn, increased interpersonal conflict. Based on the diversity management literature (Roberge and van Dick, 2010; Patrick and Kumar, 2012; Chen et al., 2021), we uncovered how inclusive HRM practices (as a top-down approach) and employee learning-oriented behaviors (as a bottom-up approach) alleviated the negative impact of workforce diversity. We found that the indirect effect of workforce diversity on interpersonal conflict through increasing negative affect was only significant when the level of inclusive HRM practices was low, or when the level of employee learning-oriented behaviors was low. In other words, when the levels of inclusive HRM practices or employee learning-oriented behaviors were high, workforce diversity would not be linked to interpersonal conflict. To conclude, our moderated mediation model on workforce diversity indicates that workforce diversity has its risks of heightening workplace interpersonal conflicts and that inclusive HRM practices initiated by organizations and learning-oriented behaviors initiated by employees themselves are two effective approaches to unlocking the potential of workforce diversity.

### Theoretical implications

Our study first enriches the workplace diversity literature (Guillaume et al., 2017; Chen et al., 2021; Wang, 2022) by empirically examining the negative consequence of workforce diversity. Our results showed that interpersonal conflict was one of the salient consequences of workforce diversity, which implies that diverse identities and backgrounds are very likely to lead to interpersonal conflict. In other words, even if diversity is beneficial to information processing and decision-making as suggested by previous diversity studies (Homan et al., 2008; Roberge and van Dick, 2010; Martin-Alcazar et al., 2012), we should not ignore the potential risk of diversity. Thus, our results also echo the diversity literature acknowledging the “double-edged” effects of diversity (Van Knippenberg et al., 2004; Van Dick et al., 2008; Wang, 2022).

Second, our study adds to the diversity management literature (Roberson, 2006; Allen et al., 2008) by providing two effective approaches to managing diversity – inclusive HRM practice and employee learning-oriented behavior. Previous diversity studies uncovered the importance of inclusion and an inclusive work environment in unlocking the benefits of diversity (Mor Barak et al., 2016; Veli Korkmaz et al., 2022). We reveal that a way to create an inclusive work environment might be implementing inclusive HRM practices including improving the procedures of fairness, openness, co-operativeness, support, and empowerment. Many previous inclusion studies emphasised the important role of leaders (e.g., inclusive leadership) in releasing the benefits of workforce diversity (Ye et al., 2019; Ashikali et al., 2020; Leroy et al., 2021). Our study adds to this line of research and suggests that in addition to leaders, the whole organization should engage in more inclusive HMR practices to decrease bias and discrimination, as well as to increase organizational justice and support. Besides, we add to the HMR literature (Okay-Somerville and Scholarios, 2019) and echo the study of Guest (2017) by indicating that inclusive HRM is a more well-being-oriented HRM practice where organizations care about the positive employment relationship and make efforts to create a fair, psychologically secure, and high-quality work environment.

Besides the top-down approach to managing workforce diversity, another important contribution we highlight is the important role of the bottom-up approach in unlocking the potential of diversity. That is, in addition to organizational practices, employees themselves should also proactively take action to capitalize on the benefits of diversity and optimize the detriments and challenges presented by diversity. Our study shows that when employees engaged in more learning-oriented behaviors, the detrimental impact of workforce diversity was weakened. Based on the categorization-elaboration model proposed by Van Knippenberg et al. (2004), our finding implies that learning-oriented behaviors can help employees to facilitate the information elaboration process and reduce the social categorization process in a diverse work environment. That is, employee learning-oriented behaviors can contribute to a better exchange of different information, ideas, and perspectives from diverse colleagues, as well as decrease misunderstandings, inaccurate judgement, and bias. This finding was in line with the study of Wang et al. (2022) indicating that individual proactive action is an important condition to gain the actual benefits of workplace diversity. This finding also aligns with the individual proactivity literature indicating that being proactive is particularly useful when the work environment is demanding and challenging (Crant, 2000; Ghitulescu, 2012). Thus, different from mainstream diversity literature uncovering organizational-level and/or team-level moderators (e.g., inclusive climate, transformational leadership, shared team goals; Muchiri and Ayoko, 2013; Nishii, 2013; Van Knippenberg et al., 2013), we reveal that personal-level moderators in the relationship between diversity and outcomes. More specifically, we reveal the importance of personal proactive *behaviors* in managing diversity rather than simply personal cognitions (e.g., openness to experience and diversity mindset) (Homan et al., 2008; Van Knippenberg et al., 2013). We argue that awareness of diversity issues might not be sufficient to lead to any changes of one’s behaivors. To gain the actual benefits and cope with the challenges presented by diversity, employees need to engage in bottom-up behaviors.

Finally, our study aimed to bring more attention of the workforce diversity issue in the Asian context. Although the diversity issue has been well noticed and somehow addressed in the western context (Shore et al., 2011), the workplace diversity phenomenon and the approaches to managing diversity are still being overlooked in many Asian organizations. Even if it is known that many Asian organizations (e.g., Chinese enterprises) do not have racial/nationality diversity in the workplace, scholars have highlighted that diversity has more attributes than just ethnicity attribute (Evans and Carson, 2005; van Knippenberg and Schippers, 2007). Our study enriches diversity management studies in the Asian context (Chen et al., 2021) by providing empirical evidence of the negative consequence of workforce diversity and by examining two approaches that can alleviate the negative impact of diversity.

### Practical implications

The practical implications of this study are also evident. First, we suggest that organizations should be aware of the negative consequence of workforce diversity besides pursuing the beneficial outcomes of workforce diversity. Our results showed that workforce diversity was positively related to interpersonal conflict *via* increasing negative affect. Thus, organizations need to think of ways to take care of interpersonal relations and coordination among diverse colleagues. Second, our results indicated that inclusive HRM practices and employee learning-oriented behaviors are two effective approaches to alleviate the negative impact of diversity. Thus, we suggest that organizations need to implement more inclusive HRM practices to unlock the potential of workforce diversity. For example, organizations need to enhance organizational justice and fairness, encourage employee participation in the decision-making process, and offer necessary support when employees feel needed (Tang et al., 2015). With these HRM practices, diverse employees can feel more psychologically safe, dare to voice their opinions, and feel engaged and energetic at work (Guest, 2017; Hur, 2020). Finally, organizations need to encourage employee learning-oriented behaviors. Organizations can empower employees with more autonomy to engage in learning-oriented activities. Research shows that the learning orientation can facilitate self-development by gaining new knowledge and expertise (Maurer, 2002) and that learning-oriented employees can proactively cope with more challenging tasks (Hirst et al., 2009). Thus, organizations should create a beneficial circumstance (e.g., a supportive learning environment; Choi and Jacobs, 2011) where employees can voluntarily learn from each other, capitalize on each other’s expertise, and decrease misunderstandings.

### Limitations and future directions

Our study also has some limitations, which will represent future directions. First, although we tried to raise awareness of the diversity issue in the Asian context, our study only examined the effects and moderators of workforce diversity in Chinese organizations. Future studies can expand our research model in other Asian countries, recognizing the level of diversity in the current workplace and enabling both top-down and bottom-up approaches to manage diversity.

Second, our study mainly provided insights into how to mitigate the negative impact of workforce diversity on employees. However, according to the diversity literature (Guillaume et al., 2017), workforce diversity also has beneficial impacts on employees (e.g., performance and well-being). Future studies can incorporate both positive impact and negative impact of workforce diversity and provide an integrative model of how to maximize the positive impact and minimize the negative impact of workforce diversity.

Third, although diversity studies have provided many insights into the effects of diversity on team-level outcomes (e.g., team cohesion, team innovation, and team conflict) and organizational-level outcomes (e.g., organizational performance and profitability) (Homberg and Bui, 2013; Shemla et al., 2016), we found that the investigation of diversity on employees, especially employee engagement/burnout and other occupational health outcomes, was relatively limited. This is important because employees’ occupational health becomes more relevant than ever before. Employees have the right to own a healthy, thriving working life. Thus, future studies can shift attention to the impact of workplace diversity on employee occupational health outcomes and how to design a stimulating job to help employees fit a diverse work environment. For example, Schulte et al. (2020) combined the job demands-resources model to examine the effect of perceived dissimilarity on work exhaustion. This is a good start and thus we recommend future studies to follow the job demands-resources framework to provide more insights into effective diversity management strategies. This can help employees to build a better person-job fit and increase job performance, as Li et al. (2021) suggested.

Fourth, it is also worthwhile to examine the (buffering) effect of diversity training on the relationship between workforce diversity and interpersonal conflict. As the diversity training literature suggests (Kalinoski et al., 2013; Bezrukova et al., 2016), diversity training can promote cognitive-based, affective-based, and skill-based learning outcomes. When employees increase their diversity awareness and diversity-related bahaviors (see, Kalinoski et al., 2013, p. 1078), they will be more likely to exchange divergent information and perspectives with one another, make use of others’ strengths, and decrease misunderstanding and bias. However, we did not measure this variable in our two-wave surveys. Thus, we believe that this can be a very important future avenue. More importantly, since prior studies found the mixed effects of diversity training (see a meta-review, Bezrukova et al., 2016), we strongly recommend future studies to develop effective diversity training to unlock more potential of diversity (especially in the Asian area). Based on the suggestions by Bezrukova et al. (2016), future studies can consider maximizing participants’ learning opportunities (e.g., combined and multi-instructional training).

Fifth, although we refer to employee learning-oriented behavior as an employee-initiated, bottom-up strategy to manage workforce diversity, we acknowledge that employee learning behaviors are trainable, and that diversity training will be an important antecedent of employee learning behaviors. For example, the diversity training literature has demonstrated that effective diversity training can increase cognitive-based, affective-based, and skill-based learning outcomes (Kalinoski et al., 2013; Bezrukova et al., 2016). Thus, we recognize diversity training as an important future research direction, in order to train employees’ different learning outcomes and increase their diversity-related beliefs and behaviors.

## Data availability statement

The raw data supporting the conclusions of this article will be made available by the authors, without undue reservation.

## Ethics statement

The studies involving human participants were reviewed and approved by Shandong Youth University of Political Science. The patients/participants provided their written informed consent to participate in this study.

## Author contributions

JL: study design, data collection, and paper writing. YZ: study design and data collection. HW: study design, paper editing, commenting, and polishing. All authors contributed to the article and approved the submitted version.

## Conflict of interest

The authors declare that the research was conducted in the absence of any commercial or financial relationships that could be construed as a potential conflict of interest.

## Publisher’s note

All claims expressed in this article are solely those of the authors and do not necessarily represent those of their affiliated organizations, or those of the publisher, the editors and the reviewers. Any product that may be evaluated in this article, or claim that may be made by its manufacturer, is not guaranteed or endorsed by the publisher.

## Footnotes

^1^where is the proportion of work group members in each of the categories on an attribute.

## Appendix

Items of inclusive HRM practice used in this study

Five-point Likert scale from 1 (very disagree) to 5 (very agree)

1. My organization is being fair in management processes and interpersonal treatment and distribution of opportunities.
2. My organization makes everyone feel the sense of belongings.
3. My organization allows everyone to keep their own uniqueness.
4. My organization includes people from diverse backgrounds and their contribution valued by the organization.
5. My organization organizes activities for team building to improve the relations and communications among employees and departments.
6. My organization provides channels for voice at each level of the organization.
7. My organization encourages employees to communicate and share within the workgroup.
8. My organization flattens the hierarchical structure to encourage employees to participate in the demographic decision.
9. My organization offers inclusion management training class for supervisors and managers.
10. My organization implements open and equal recruitment procedures.
11. My organization advises supervisors to tolerate subordinates’ mistakes.
